# Sprayable phage-cocktail hydrogel promotes diabetic wound healing through macrophage M2 polarization and restoring SP and CGRP signaling axis

**DOI:** 10.3389/fbioe.2026.1789996

**Published:** 2026-10-12

**Authors:** Han-Shiang Shiue, Sheng-Jie Shiue, Hsin-Yi Lin, Ming-Shun Wu

**Affiliations:** 1 Division of Gastroenterology, Department of Internal Medicine, Wan Fang Hospital, Taipei Medical University, Taipei, Taiwan; 2 International Ph.D. Program in Cell Therapy and Regenerative Medicine, College of Medicine, Taipei Medical University, Taipei, Taiwan; 3 Graduate Institute of Chemical Engineering, National Taipei University of Technology, Taipei, Taiwan; 4 Graduate Institute of Biochemical and Biomedical Engineering, National Taipei University of Technology, Taipei, Taiwan; 5 Division of Gastroenterology and Hepatology, Department of Internal Medicine, School of Medicine, College of Medicine, Taipei Medical University, Taipei, Taiwan; 6 International PhD Program in Medicine, College of Medicine, Taipei Medical University, Taipei, Taiwan; 7 TMU Research Center for Digestive Medicine, Taipei Medical University, Taipei, Taiwan

**Keywords:** bacteriophage therapy, diabetes, macrophage polarization, neuroimmune modulation, wound healing

## Abstract

**Aims:**

Chronic diabetic wounds often fail to heal due to persistent infections and dysregulated immune responses. This study developed a sprayable phage-cocktail hydrogel, a hybrid living material, to provide simultaneous infection control and neuroimmune modulation.

**Methods:**

A phage-cocktail dressing was formulated using three bacteriophage strains targeting *Salmonella enterica, Escherichia coli, and Pseudomonas aeruginosa*. These bacteriophages were mixed with a 1% sodium alginate solution to create a hands-free spray-produced dressing. The dressing was evaluated for antibacterial activity, cytocompatibility with 3T3 fibroblasts, and efficacy in healing sterile full-thickness skin wounds in *db/db* mice. The skin wound recovery, macrophage polarization, calcitonin gene-related peptide (CGRP) and substance P (SP) expression were assessed through histological analysis.

**Results:**

The dressing achieved approximately 90% antibacterial efficacy while maintaining excellent skin cell cytocompatibility. In db/db mice, the treatment significantly accelerated wound closure (94.2% by day 14), prevented ulceration, and enhanced tissue regeneration. Mechanistic analysis revealed that the dressing induced M2 macrophage polarization and modulated neuropeptide signaling. Specifically, epidermal CGRP sustained M2 macrophages to promote re-epithelialization, while dermal SP facilitated extracellular matrix (ECM) remodeling.

**Conclusion:**

This sprayable hydrogel integrates antimicrobial efficacy with neuroimmune modulation to accelerate diabetic wound healing. By modulating pathways associated with the SP/CGRP signaling axis, it supports functional ECM remodeling and re-epithelialization while mitigating dermal fibrosis. This layer-specific regulation provides a potent therapeutic framework for achieving regenerative tissue repair in chronic ulcers.

## Introduction

Wound infections and impaired healing are serious complications affecting approximately 15% of the global diabetic population, nearly 150 million people. Alarmingly, about half of these cases progress to amputation (Armstrong et al., 2017; Meloni et al., 2018). Diabetes impairs immune function and promotes chronic inflammation, oxidative stress, neuropathy, and defective cell migration, making wounds more vulnerable to persistent infections. The pathogens such as *Pseudomonas aeruginosa*, *Staphylococcus aureus*, and *Escherichia coli* are often implicated in diabetic wound infections, where they form resilient biofilms that significantly hinder treatment efficacy and contribute to poor clinical outcomes (Akash et al., 2020; Burgess et al., 2021; Rodríguez-Rodríguez et al., 2022). For diabetic patients, wound infections not only delay healing but can also become life-threatening. Hence, effective disinfection and antimicrobial prevention are crucial for reducing the risk of infection.

Bacteriophages (phages) are gaining attention as effective treatments for antibiotic-resistant infections (Hibstu et al., 2022; Strathdee et al., 2023). When integrated into wound dressings, particularly hydrogels, phage therapy enhances chronic wound healing (Sanjanwala et al., 2022; Shiue et al., 2024). Clinical studies report a 70% recovery rate, with marked improvement in 25% of patients using phage-loaded hydrogels (Markoishvili et al., 2002). Additionally, electrospun polycaprolactone polymers embedded with phages have shown over 90% antibacterial efficacy without adverse effects in rabbit models (Nogueira et al., 2017; Cheng et al., 2018), highlighting their potential for safe and efficient therapeutic applications in wound care.

Macrophages, known for their plasticity, are central to wound healing, transitioning from the pro-inflammatory M1 phenotype to the pro-healing M2 phenotype as the healing process progresses (Jończyk-Matysiak et al., 2017). Dysregulated macrophage responses, often seen in diabetic wounds, impair healing (Sharifiaghdam et al., 2022). Neuropeptides such as calcitonin gene-related peptide (CGRP) and substance P (SP) play critical roles in neuroimmune communication and wound healing. CGRP acts on immune cells like neutrophils and macrophages, suppressing their overactivation, promoting efferocytosis, and shifting macrophages toward a reparative phenotype (Lu et al., 2024). In diabetic conditions, CGRP and SP expression is often downregulated, contributing to impaired healing (Yang et al., 2022). A key focus of this study is to elucidate how the phage-cocktail hydrogel dressing influences macrophage phenotypes and regulates the expression of CGRP and SP in obese-diabetic wound healing.

Calcium alginate, derived from seaweed, crosslinks with calcium or magnesium ions to form a biocompatible, biodegradable, non-toxic, and cost-effective hydrogel dressing (Hsu et al., 2020; Ahmad Raus et al., 2021). Upon application, the alginate hydrogel film exhibits high structural integrity with a Young’s modulus of 4,602 MPa (Contardi et al., 2022). Additionally, once a Ca-alginate hydrogel is placed on a physiological environment such as a wound bed, which is rich in Na^+^ and K^+^, Ca ions are displaced by these monovalent ions and alginate hydrogel will liquidize (Peng et al., 2012; Contardi et al., 2022). This dynamic ion exchange prevents tissue sticking and pain during dressing changes, with saline facilitating easy removal (Varaprasad et al., 2020). Recent advanced hydrogels excel in refractory wound management, yet conventional dressings relying on broad-spectrum antibiotics may promote bacterial resistance and disrupt skin microbiome homeostasis, ultimately hindering wound healing. Meanwhile, bioactive advanced cell-free therapies fail against biofilms (Savitri et al., 2022; Yang et al., 2022; Lin et al., 2025). To address both typical and atypical diabetic wound infections, including opportunistic *Salmonella enterica* driven by impaired immunity and vascular disease, we developed a sprayable alginate hydrogel loaded with a broad-spectrum bacteriophage cocktail targeting *E*. *coli*, *P*. *aeruginosa*, and *S. enterica* (Chiao et al., 2016; Dilhari et al., 2020; Kim and Choi, 2025). A phage cocktail targeting these strains was mixed with sodium alginate and sprayed onto a collector, forming an adhesive film, simulating a hands-free application method. Unlike fixed-size, non-antimicrobial PU dressings, this format conforms to wound shape and allows continuous wound monitoring.

Given the global co-prevalence of obesity and type 2 diabetes mellitus (T2DM) (Khan et al., 2023; Muthiah et al., 2023), we used obese-diabetic db/db C57BL/6 mice to model this comorbidity. We hypothesized that the phage-cocktail hydrogel would exhibit antimicrobial activity, promote wound healing, and favorably influence neuroimmune interactions involving macrophages, CGRP, and SP. Notably, recent landmark studies have established that CGRP sensory neurons naturally promote macrophage M2 polarization and tissue healing (Lu et al., 2024); however, diabetic neuropathy paralyzes the healing CGRP/SP axis, arresting wounds in inflammation (Feldman et al., 2019; Yang et al., 2022). In this study, we investigated and demonstrated for the first time that this sprayable phage-cocktail hydrogel modulates the layer-specific spatial dynamics of epidermal CGRP and dermal SP expression, which subsequently drives reparative M2 macrophage polarization within the diabetic wound microenvironment.

## Material and methods

### Phage purification and characterization

*Escherichia coli* (BCRC 43064), *S. enterica* (BCRC 10747), and *P. aeruginosa* (BCRC 11633) were grown in 3% (w/v) tryptone soy broth (TSB) at 37 °C. Phages Ph(E), Ph(S), and Ph(P) were isolated from Taipei Dihua wastewater via double-layer agar assays. After 0.22-μm filtration and overnight host enrichment (OD600 = 0.4), phages were purified through serial isolation and stored in TSB at 4 °C. Titers (PFU/mL) were measured by drop-plating. Morphology and dimensions (n = 3) were analyzed using FE-TEM (JEM-2100F) with 2% uranyl acetate and ImageJ. Host-specific lytic activity was confirmed by plaque assays (Shiue et al., 2024).

### Phage-cocktail hydrogel dressing sample production

Sterile 10% (w/v) NaCl was incorporated into 2% (w/v) alginate (450–550 kDa, Acros) to modulate ionic strength, thereby optimizing the viscosity and mechanical integrity of the sprayable dressing. The alginate solution was mixed 1:1 with phages to a final concentration of 1 × 10^8^ PFU/mL, exceeding typical therapeutic titers (1 × 10^6^−1 × 10^7^ PFU/mL). Calcium chloride (CaCl_2_) served as the ionic cross-linking agent to induce gelation and film formation. Sprayable hydrogel dressings were fabricated and systematically evaluated by combining various sodium alginate concentrations (1.0%, 1.5%, and 1.8% w/v) with CaCl_2_ concentrations (10% and 30% w/v), as summarized in Table 1. For film production (Figure 1), solutions were loaded into a sterile PT-29 sprayer (Auarita) within a clean bench. Compressed air was 0.22-μm filtered before spraying onto skin-mimicking IPM silicone. The other spray deposition parameters, including target distances (25 cm or 35 cm) and spray cycles (1 or 2 cycles), along with other operating settings (Table 1), were modulated to optimize film homogeneity, durability, and structural integrity. Film dimensions were determined from four independent samples (n = 4), with each sample measured in triplicate and averaged; thickness was determined from the thickness differential of the IPM silicone substrate before and after spray deposition (T_film_ = T_total_ – T_substrate_). Measurements were conducted using a digital vernier caliper (resolution: 0.01 mm), with readings averaged across the center and edge regions (n = 4).

**TABLE 1:** Diameters and thickness of sprayed hydrogel films made with different parameters.

L (cm)	Alginate concentration (w/v%)	Ca(II) concentration (w/v%)	Diameter (cm)	Thickness (mm)
25	1.0	10	7.13 ± 0.37	0.08 ± 0.03
		30	7.52 ± 0.72	0.08 ± 0.03
	1.5	10	7.18 ± 0.83	0.32 ± 0.08
		30	7.30 ± 0.68	0.27 ± 0.04
	1.8	10	6.49 ± 0.93	0.55 ± 0.12
		30	6.27 ± 0.34	0.61 ± 0.12
35	1.0	10	8.89 ± 0.65	0.05 ± 0.0
		30	8.77 ± 0.39	0.05 ± 0.0
	1.5	10	8.61 ± 0.45	0.13 ± 0.02
		30	8.83 ± 0.69	0.14 ± 0.04
	1.8	10	7.82 ± 0.39	0.26 ± 0.03
		30	7.98 ± 0.45	0.24 ± 0.03

Alginate concentration (w/v%)	Ca(II) concentration (w/v%)	Number of spray	Diameter (cm)	Thickness (mm)
1.0	10	1	6.93 ± 0.21	0.18 ± 0.07
		2	7.97 ± 0.24	0.27 ± 0.05
1.5		1	6.46 ± 0.31	0.44 ± 0.10
		2	7.16 ± 0.24	0.93 ± 0.13
1.8		1	5.92 ± 0.34	0.69 ± 0.08
		2	6.12 ± 0.28	1.08 ± 0.27

**FIGURE 1:**
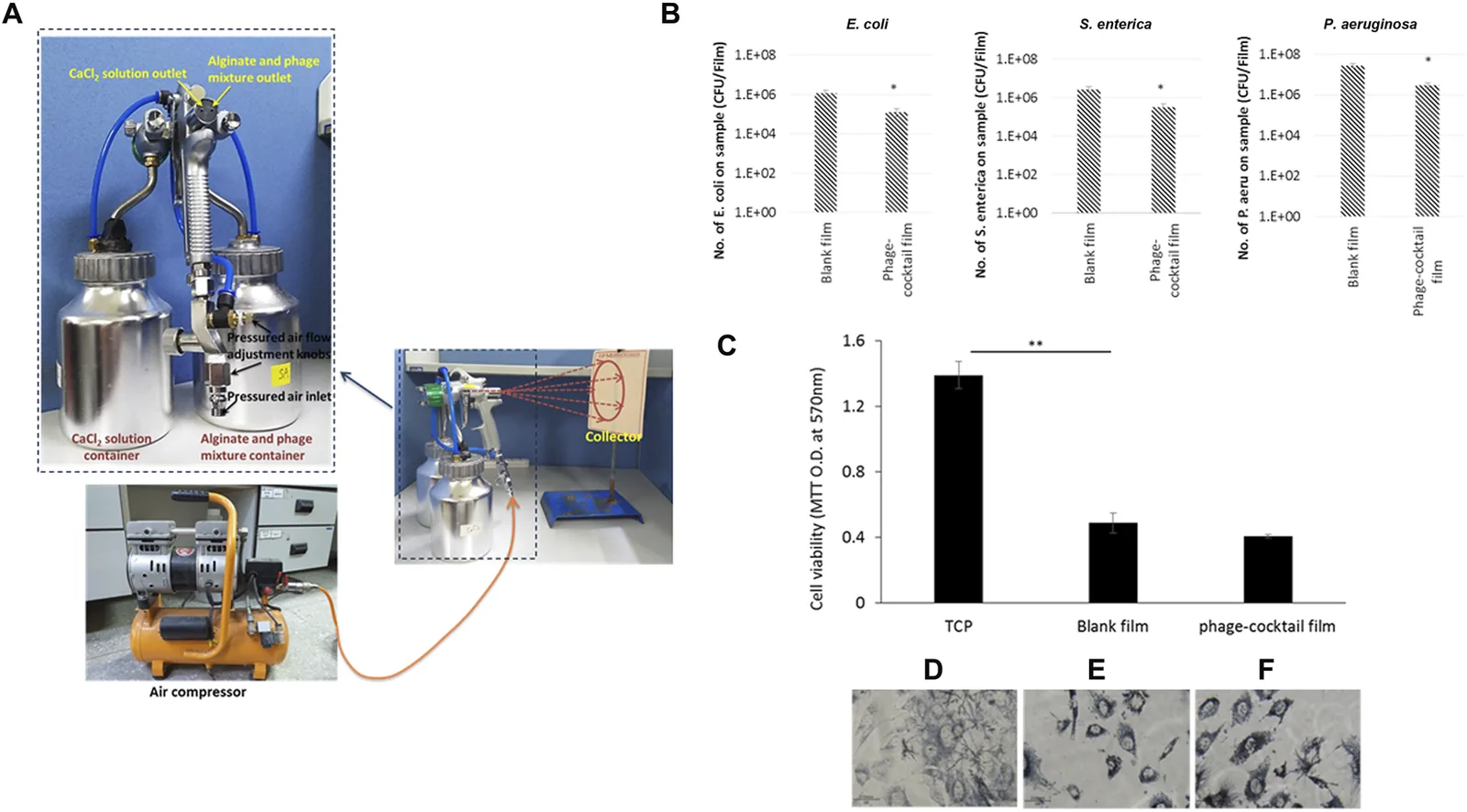
The spraying apparatus, antibacterial efficacy, and skin cell biocompatibility of phage-cocktail film. **(A)** Illustration of the spraying apparatus for phage-cocktail film production. **(B)** Antibacterial efficacy of phage-cocktail film 24 h after bacteria seeding. *: p < 0.001 compared to the blank film. **(C)** Skin cell biocompatibility via MTT and **(D–F)** cell morphology on TCP, blank, and phage films 24 h after cell seeding.

### Antibacterial test

The antibacterial test was measured using protocols based on ISO 22196: test for antimicrobial activity of plastics, with modifications in our previous study (Shiue et al., 2024). *Escherichia coli*, *S. enterica*, and *P. aeruginosa* were cultured in 3% (w/v) TSB at 37 °C. Assays were performed in sterile 24-well plates with control (plain) and test (phage-loaded) hydrogels (n = 4 per group). Each was inoculated with 100 µL of bacterial suspension (OD600 = 0.2, about 1.6 × 10^7^ CFU/mL), with baseline counts determined before incubation. After 24 h at 37 °C, surviving bacteria were recovered using 1 mL sterile water and quantified via pour plating (Shiue et al., 2024).

$$\text{Antibacterial rate}=\left(\text{number of bacteria on control}\;-\;\text{number of bacteria on film with bacteriophage}\right)/\left(\text{number of bacteria on control}\right)\times 100\%$$

### Skin cell biocompatibility

3T3 fibroblasts (BCRC, Taiwan) were seeded at 5 × 10^4^ cells per well in 24-well plates in supplemented DMEM (Gibco) and incubated at 37 °C with 5% CO_2_ for 24 h. Cell viability (n = 4) was assessed via MTT assay: media were replaced with 1 mg/mL MTT solution for 4 h, followed by formazan dissolution in 600 μL isopropanol. Absorbance was recorded at 470 nm using a Spectrostar Nano reader (BMG, Germany).

### Skin wound recovery in obese-diabetic mice

All animal experiments were approved by the Taipei Medical University-Wan Fang Hospital Committee of Experimental Animal Care and Use (approval No. WAN-LAC-112–10) and conducted in accordance with relevant guidelines. Obese-diabetic db/db C57BL/6 mice (Bio LASCO, Taiwan) were used (Wei et al., 2020). Obese-diabetic mice (blood Glucose >300 mg/dL) were randomized into control, blank dressing, or phage-cocktail groups (n = 6 per group). Prior to the surgical procedure, mice were anesthetized via intraperitoneal injection of a mixture of ketamine (100 mg/kg) and xylazine (10 mg/kg). Dorsal 6-mm full-thickness wounds were created, sprayed with PBS, blank dressing, or phage-cocktail dressing, and covered with Tegaderm films and elastic bandages. Carprofen (0.5 mg/100 g SC/day) was administered for 3 days post-surgery. Wound healing was monitored on days 0, 3, 7, and 14 and analyzed using ImageJ. On day 14, mice were euthanized by gradual CO_2_ inhalation with a chamber displacement rate of 30%–70% volume/min in accordance with institutional animal welfare guidelines. Wound tissues were subsequently harvested for immunohistochemistry (IHC) analysis (Wei et al., 2020; Shiue et al., 2024).

### Histological analysis and wound healing scoring

Histological analysis followed established protocols. Skin samples were fixed in 3.7% formaldehyde for 2 days, dehydrated in graded ethanol, cleared with xylene, and embedded in paraffin. Sections (6 μm) were stained with H&E and Masson’s trichrome. Wound healing was semi-quantitatively scored (0–4) based on re-epithelialization: 0 (cut edges), 1 (<50% migration), 2 (≥50%), 3 (bridged), 4 (keratinized), assessed blindly by independent reviewers (Ismail and Ishak, 2022). Immunofluorescence identified M1 and M2 macrophages using rabbit anti-pSTAT1 (1:500) and anti-CD163 (1:500). Immunohistochemistry (IHC) detected neuropeptides using rabbit anti-CGRP (1:1,000) and anti-substance P (1:500). For immunofluorescence analysis, the percentage of fluorescent areas were quantified using ImageJ, while IHC data were evaluated using the H-score by pathologists (McCarty et al., 1985; Gylten et al., 2025). Average scores were calculated for statistical comparisons between treatment groups.

### Statistical analysis

The values within the results section were expressed as means ± standard deviation. One way ANOVA with the Tukey’s *post hoc* test or Student’s t-test were used to determine differences between different groups. Values of p < 0.05 were considered significant.

## Results

### Optimization of sprayable phage-cocktail hydrogel dressing production

Sprayable hydrogel optimization results are summarized in Table 1. Increasing the nozzle-to-collector distance from 25 cm to 35 cm enlarged sample diameter but reduced thickness. Diameter was unaffected by calcium chloride concentration, while thickness increased with alginate concentration and spray count. At 1.8% alginate, the solution became overly viscous, yielding uneven surfaces and films prone to central shrinkage and detachment. To ensure film stability on skin, 1% sodium alginate was selected. Optimal conditions, 35 cm spray distance, 1% alginate, 10% calcium chloride, and one spray, produced uniform, manageable films. Final samples (1 cm diameter) were punched and used for *in vitro* testing in sterile 24-well TCPs.

### Antibacterial ability of the phage-cocktail hydrogel dressing

The sprayable phage-cocktail hydrogel dressing significantly inhibited bacterial growth (p < 0.01) within 24 h, reducing bacterial counts by nearly 90% compared to the phage-free control (Figure 1B). Antibacterial rates were 89.2% for *E. coli*, 88.4% for *Salmonella*, and 89.1% for *P. aeruginosa*, respectively, confirming antibacterial efficacy was maintained after 24 h of exposure and demonstrating the successful incorporation and retained lytic activity of phages within the hydrogel dressing.

### Sprayable phage-cocktail dressing shows good skin cell biocompatibility and low-adherent properties

As shown in Figure 1C, cell viability on the phage-cocktail hydrogel was comparable to the blank hydrogel, indicating no cytotoxicity. Furthermore, after normalizing the MTT data to cell counts (using image analysis), no significant differences in viability were observed among the phage-cocktail hydrogel, blank hydrogel, and TCP controls (data not shown). Due to alginate’s lack of cell-binding receptors, skin cells adhered poorly, clustering rather than spreading (Figures 1C–E). The low-adherent nature of the alginate-based dressing suggests a clinical benefit in enabling atraumatic removal. The phage cocktail did not inhibit cell proliferation, as shown by the comparison of Figures 1C,E,F.

### The phage-cocktail dressing effectively enhances wound closure and prevents ulceration during the wound healing process in obese-diabetes mice

In the phage group, noticeable exudation, redness, and swelling were observed from day 3 to day 7 post-injury (Figure 2A). However, no significant ulceration occurred during the treatment period. On day 3, the wound healing rate in the phage group exceeded 50% (51.5% ± 4.3%), significantly surpassing the control (20.3% ± 2.9%) (Figure 2B). By day 14, the wounds were almost completely healed with a healing rate of 94.2% ± 0.6%. In contrast, the control with PBS group exhibited substantial ulceration, inflammation, and granuloma on day 14, with a lower healing rate of 60.0% ± 3.9% (Figure 2B). In the blank dressing group, noticeable ulceration, exudation, redness, and swelling were observed from day 3 to day 7 post-injury (Figure 2A). By day 14, the wound healed with a healing rate of 83.1% ± 4.5% and there was obvious slight redness, swelling and thin scabbing.

**FIGURE 2:**
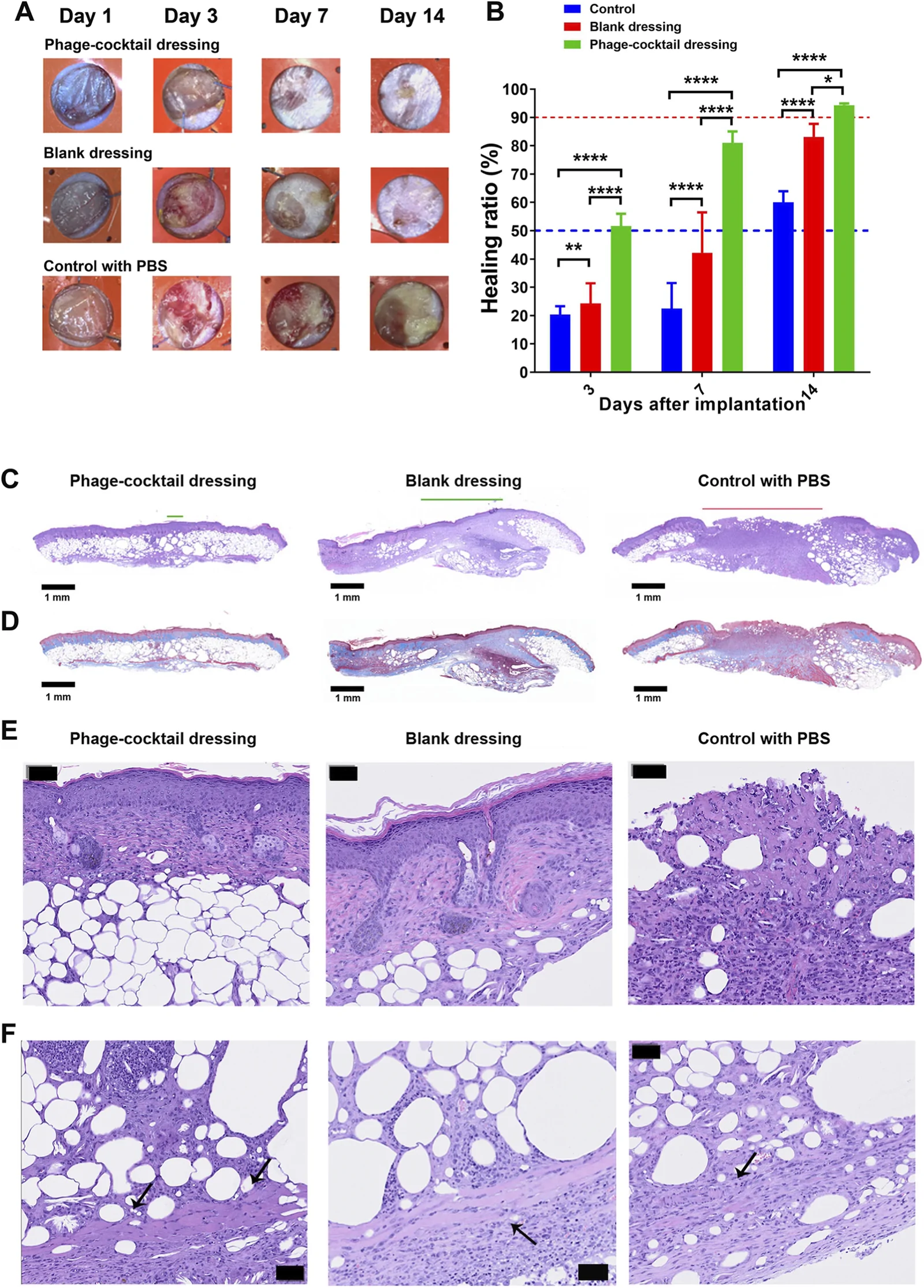
Phage-cocktail dressing accelerates wound healing in diabetic mice with full-thickness skin wound. **(A)** Wound progression was monitored at 0, 3, 7, and 14 days. **(B)** Healing ratios improved with both dressings, notably >90% in the phage-cocktail group. Red/green lines mark wound/scar thresholds; scale bars: 50 μm. (*p < 0.5; **p < 0.01; ****p < 0.0001). **(C)** Representative Hematoxylin and eosin (H&E) stained and **(D)** Masson’s trichrome (MT) stained skin wound sections on day 14 (scale bars = 1 mm). **(E)** High-magnification histological views of the wound areas showing re-epithelialization, tissue structure, and **(F)** the regenerated panniculus carnosus muscle layer (indicated by arrows).

### The histological analysis and semi-quantitative evaluation highlight the potential of the phage cocktail dressing in promoting re-epithelialization progress

Histological analysis revealed significant differences in wound repair among treatment groups after 14 days (Figure 2C). The phage group showed the most complete restoration, with full re-epithelialization and regeneration of the epidermis, dermis, adipose tissue, and panniculus carnosus muscle layers (Figures 2E,F). Masson’s trichrome staining confirmed enhanced collagen deposition (Figure 2D). This group also exhibited well-organized epidermal layers and normal dermal structures, including hair follicles and sebaceous glands (Figure 3A). In contrast, the blank dressing group showed partial structural disorganization and inflammation, while the control group exhibited severe fibrosis, inflammation and incomplete muscle repair. The phage group exhibited smaller scar areas and more organized epidermal stratification across the basal, spinous, granular, and cornified layers compared to the other two groups (Figure 3B). Semi-quantitative scoring further confirmed superior re-epithelialization (Figure 3C). Overall, the phage-cocktail dressing markedly enhanced skin regeneration and reduced inflammation.

**FIGURE 3:**
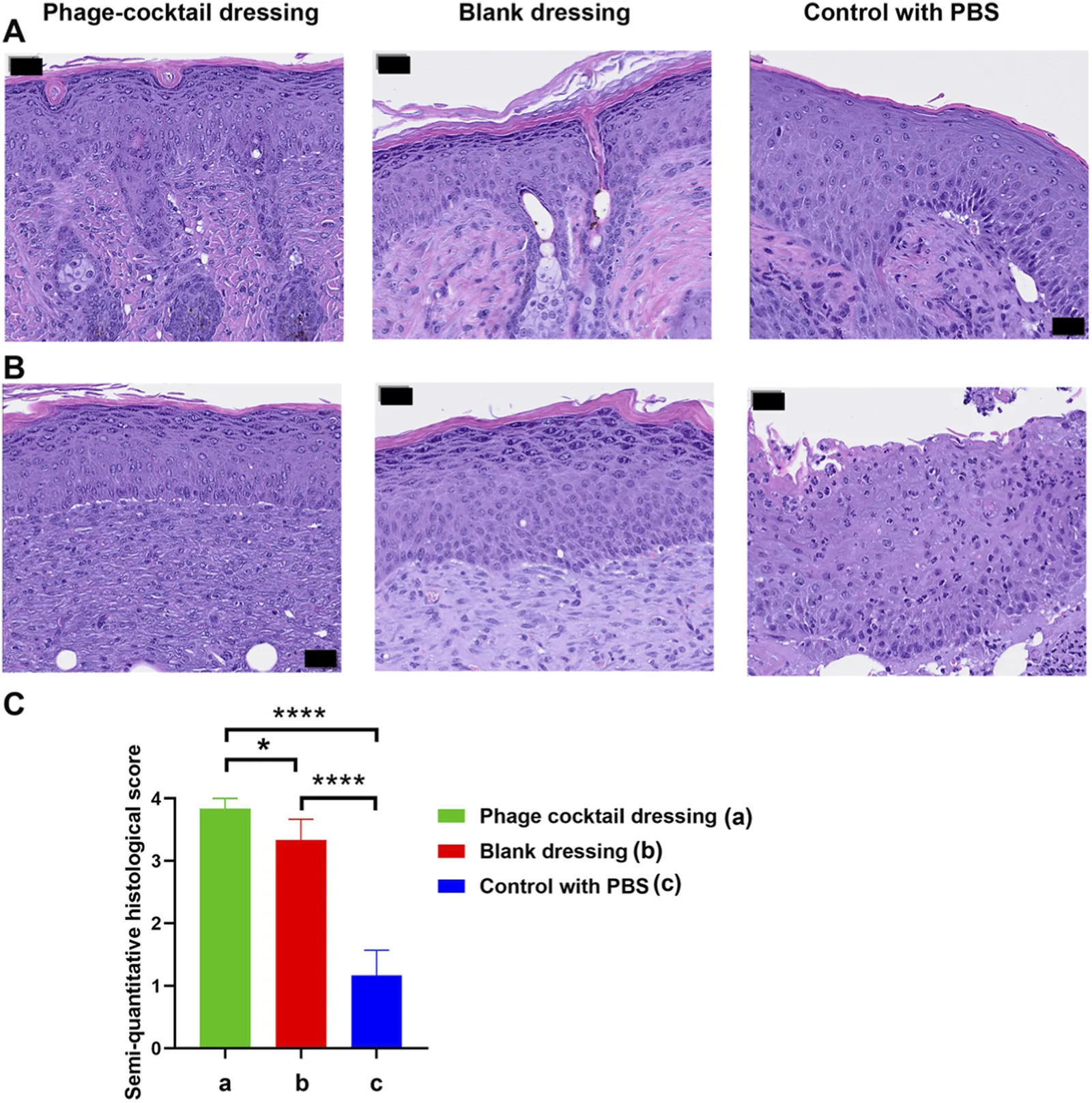
Semi-quantitative histological scorings show the most complete epithelialization in phage-cocktail dressing group **(A,B)** H&E-stained and **(C)** Semi-quantitative histological scorings showed the histological changes in of epidermis and dermis at post-wounding day 14. Scale bars: 20 μm.

### The phage-cocktail dressing enhances the macrophage M2 polarization in regenerated wound tissue

To evaluate the effect of the phage-cocktail dressing on the wound immune microenvironment, we performed immunofluorescence analysis at day 14 post-wounding to characterize macrophage polarization. In control groups, the wound tissue exhibited a dominant infiltration of M1 macrophages (pSTAT1 colocalized with CD68), indicating a persistent pro-inflammatory state (Figure 4A). In contrast, treatment with the phage-cocktail dressing significantly shifted the macrophage population toward an M2 phenotype (cMAF colocalized with CD163), suggesting a successful transition from the inflammatory phase to the reparative phase (Figure 4B). Spatial analysis revealed that phage-cocktail dressing increased M2 macrophage density throughout all skin layers, while control wounds remained dominated by M1 macrophages. (Figure 4C). In the zoomed-in image, M2 macrophages within the epidermis were primarily localized to the granular layer, whereas M1 and transitioning M1-to-M2 macrophages were predominantly observed in the spinous and basal layers in phage-cocktail dressing group, whereas control wounds showed abundant M1 macrophages distributed throughout all skin layers (Figure 4D). The phage-cocktail dressing significantly decreased pro-inflammatory M1 macrophages while increasing anti-inflammatory M2 macrophages compared to the blank dressing and PBS control groups (Figure 4E, *p* < 0.01).

**FIGURE 4:**
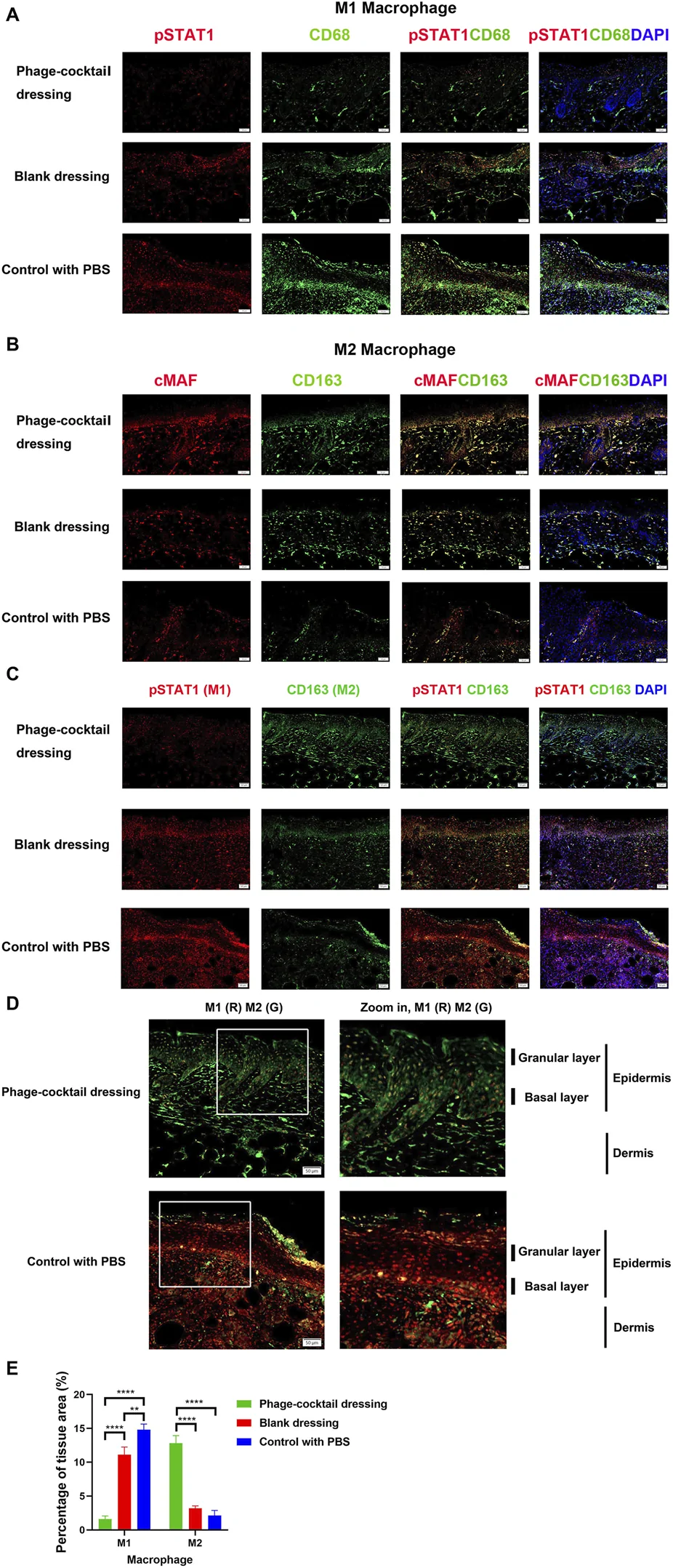
Phage-cocktail dressing enhances macrophage M2 polarization. **(A)** Control groups exhibited widespread infiltration of M1 macrophages (M1 markers: pSTAT1, red; CD68, green) across the wound bed. **(B)** Phage-cocktail dressing markedly enhanced M2 macrophage polarization (M2 markers: cMAF, red; CD163, green). **(C)** Phage-cocktail dressing increased epidermal M2 macrophages, while controls showed abundant M1 macrophages throughout skin layers. **(D)** In the zoomed-in image of epidermis, M2 macrophages localized to the granular layer, while M1 and transitioning M1-to-M2 macrophages occupied lower epidermal layers. Immunofluorescence analysis at day 14 post-wounding showed macrophage polarization in wound tissue: M1 (pSTAT1, red), M2 (CD163, green), and nuclei (DAPI, blue). Scale bars: 50 μm. **(E)** Quantification of M1 and M2 macrophage in wound tissue. Results are expressed as the percentage of tissue area positive for M1 or M2 macrophage in skin tissue at day 14 post-injury. (n = 10; **p < 0.01; ****p < 0.0001).

### Phage-cocktail dressing is associated with altered spatial dynamics of SP and CGRP expression

At day 14 post-wounding, SP and CGRP expression in the epidermis was elevated in the phage group (Figure 5). Their localization varied across epidermal layers: CGRP was primarily detected in the granular layer, where M2 macrophages are abundant, while SP was mainly distributed in the spinous and basal layers, regions associated with M1 or transitioning M1-to-M2 macrophages. In the dermis, CGRP was predominantly localized to the papillary dermis and around nerve endings, whereas SP was more diffusely distributed throughout both the papillary and reticular dermis, co-localizing with activated fibroblasts. In contrast, control groups exhibited minimal SP and CGRP expressions within the disorganized epidermis, while dermal CGRP remained detectable and SP was largely absent or restricted to the wound margins (Figure 5A). The phage-cocktail dressing significantly increased SP expression (epidermis/dermis) and epidermal CGRP, while significantly suppressing dermal CGRP compared to the blank dressing and PBS controls (Figure 5B, p < 0.05).

**FIGURE 5:**
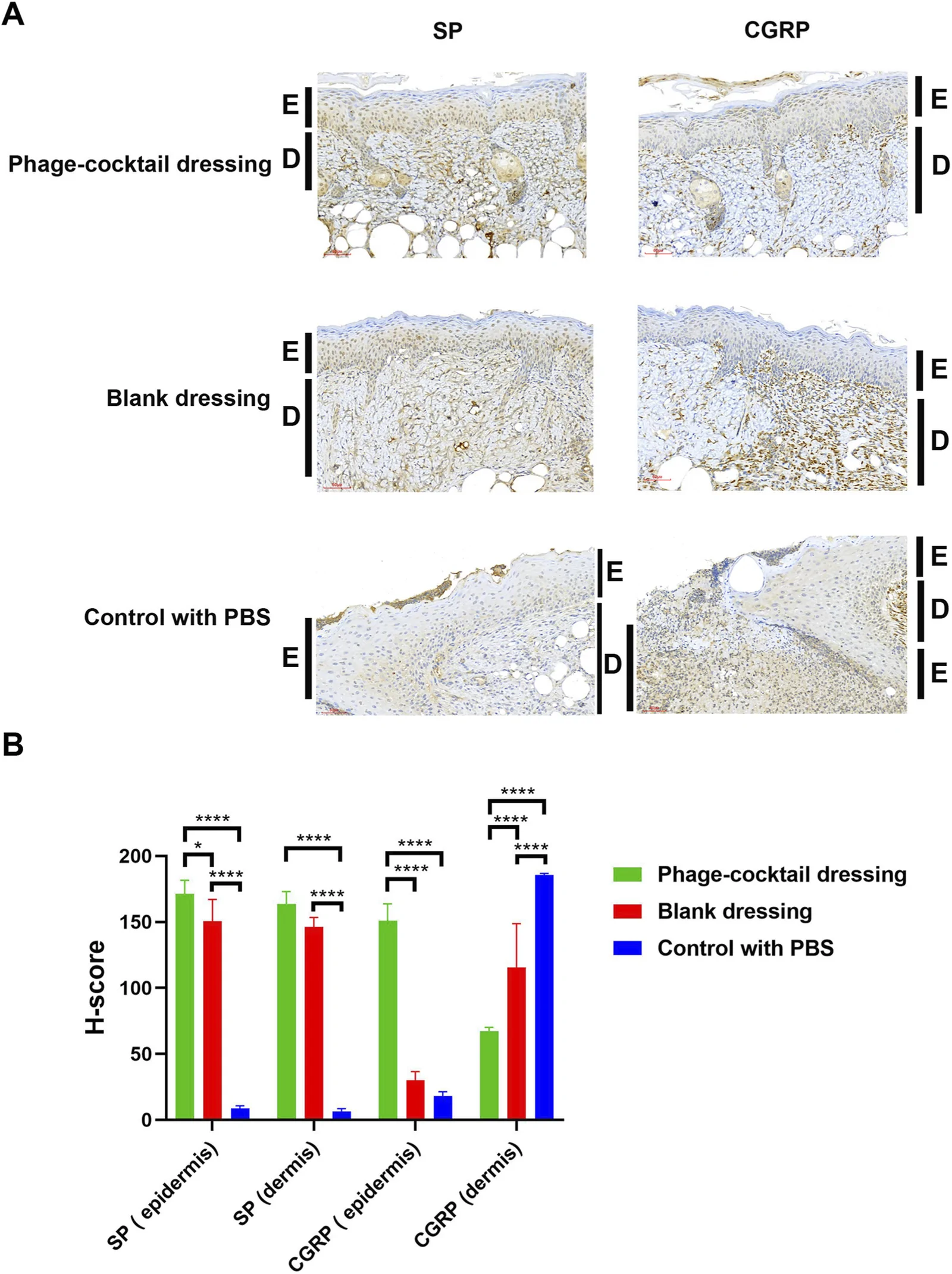
Phage-cocktail dressing enhances substance P expression in epidermis and dermis, and upregulates calcitonin gene-related peptide in the epidermis. **(A)** Immunohistochemistry revealed the expression of neuropeptides SP and CGRP in wound tissue. In epidermis region of the phage-cocktail dressing group, CGRP was primarily localized to the granular layer of the epidermis, whereas SP was mainly distributed in the spinous and basal layers. E was epidermis and D was dermis. Scale bars: 60 μm. **(B)** Quantification of CGRP and SP in wound tissue by H-score. (n = 4; *p < 0.05; ****p < 0.0001).

### Distinct spatial distribution of SP and CGRP correlates with dermal remodeling and fibrotic scar formation

In Figure 6A, histological evaluation using H&E and Masson’s trichrome staining on day 14 post-wounding identified clear morphological distinctions between scar and non-scar regions. In Figures 6B,C, IHC analysis revealed a divergent expression pattern for SP and CGRP. SP expression was broadly distributed throughout the non-scar dermis and positively correlated with soft extracellular matrix (ECM) remodeling. In contrast, CGRP was predominantly localized within scar tissue, where its expression was linked to the formation of fibrotic scars. Notably, enrichment of CGRP, rather than SP, was specifically observed in impaired wound areas, suggesting that these two neuropeptides may play distinct roles in the transition from physiological remodeling to pathological fibrosis during dermal healing. Scar tissues exhibited significantly elevated CGRP expression, but significantly reduced SP expression compared to non-scar tissues (Figure 6B, p < 0.05).

**FIGURE 6:**
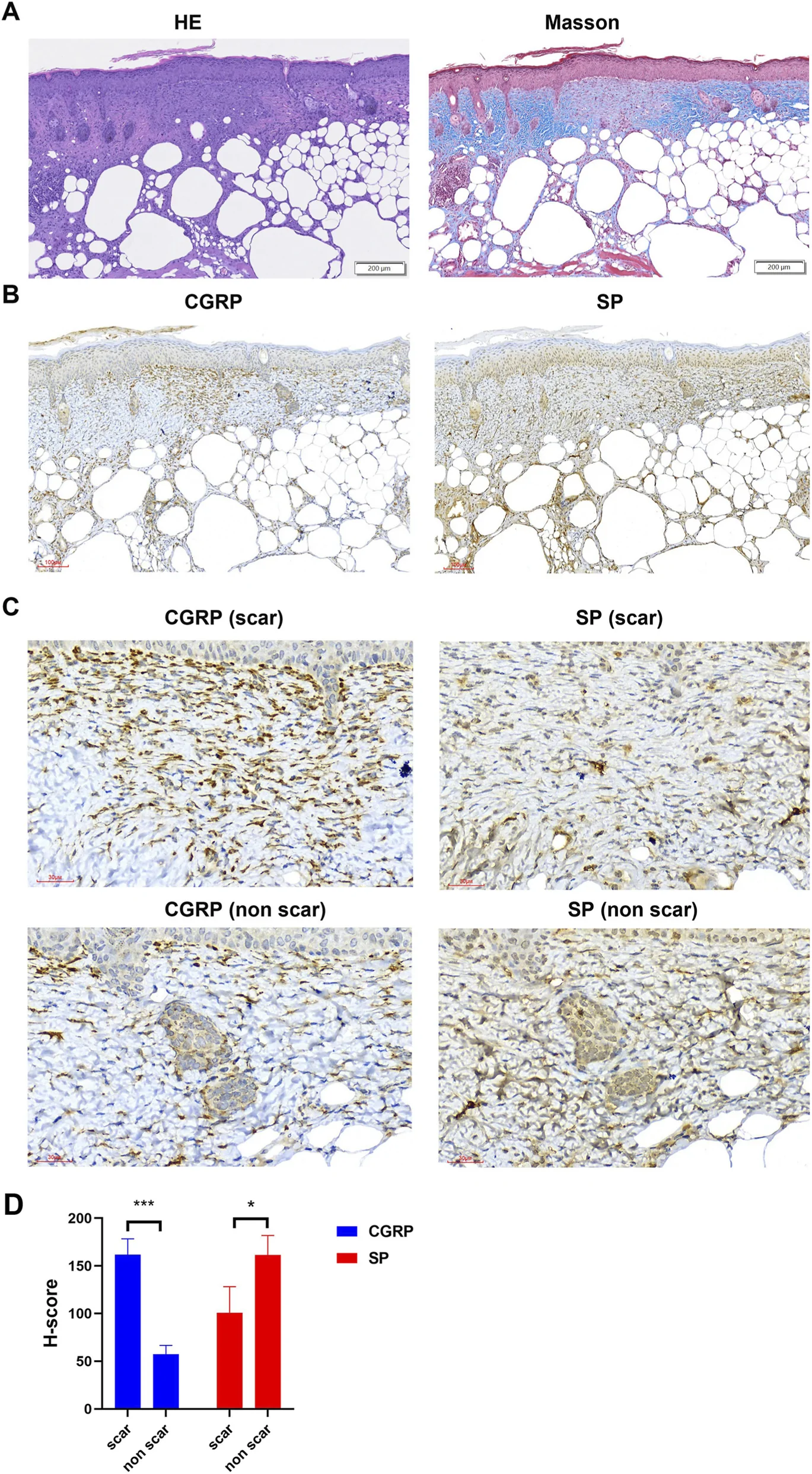
CGRP was associated with scar formation, and SP was associated with soft ECM production. **(A)** H&E-staining and MT-staining showed the histological changes in scar region and non-scar region at post-wounding day 14. **(B,C)** The IHC staining showed the expression of CGRP and SP in scar region and non-scar region in dermis. IHC showed that CGRP expression in the epidermis was independent of scar formation but was predominantly localized to scar tissue in the dermis. Scale bars: **(A)** 200 μm; **(B)** 100 μm and **(C)** 30 μm. **(D)** Quantification of CGRP and SP in IHC staining by H-score. (n = 3; *p < 0.05; ***p < 0.001).

## Discussion

This study demonstrates the therapeutic potential of the sprayable phage-cocktail hydrogel dressing for accelerating wound healing and modulating immune responses under diabetic conditions. Developed as a sprayable formulation, the optimal dressing, comprising 1% sodium alginate and 10% calcium chloride applied from a 35 cm distance, forms uniform, flexible films (Table 1). The hydrogel exhibited strong antibacterial effects, reducing *E. coli*, *Salmonella*, and *P. aeruginosa* populations by approximately 90% (Figure 1B). It was biocompatible, non-toxic to skin cells, and minimally adhesive, allowing painless removal (Figures 1C–F). Beyond its physical properties, the calcium alginate component enhances hemostasis by releasing calcium ions that accelerate the coagulation cascade (Subramaniam et al., 2021; Xie et al., 2022).

The success of this dressing lies in the synergy between the alginate matrix and the phage cocktail. Alginate promotes healing through its biocompatibility and superior moisture retention. By forming a hydrophilic gel, it manages to exudate and maintain an environment essential for re-epithelialization. This versatile matrix protects the wound bed and stabilizes the microenvironment, facilitating tissue regeneration and significant wound closure even in its unmodified form (Varaprasad et al., 2020; Froelich et al., 2023) (Figure 2, blank dressing). However, it remains a passive barrier. The specific contribution of the phage cocktail is the addition of an active antimicrobial defense. This transforms the dressing from a supportive microenvironment into a therapeutic system capable of directly eliminating bacteria and overcoming the infection-related delays common in chronic wounds (Figure 2, phage-cocktail dressing).

In obese-diabetic mice, the phage-cocktail dressing significantly accelerated wound closure (94.2% by day 14), reduced ulceration, and improved tissue regeneration (Figures 2A,B). Histological analysis confirmed enhanced re-epithelialization, organized collagen deposition, and restored skin architecture (Figure 3). Mechanistically, the dressing promoted M2 macrophage polarization (Figure 4), thereby supporting an anti-inflammatory microenvironment. Notably, the treatment upregulated SP to drive the macrophage M1-to-M2 transition and upregulated CGRP to sustain M2 macrophages within the epidermis to facilitate re-epithelialization; meanwhile, dermal SP was found to promote ECM remodeling (Figure 5). Interestingly, we observed an unexpected divergence in neuropeptide function: SP expression correlated with favorable soft ECM remodeling, whereas CGRP expression in the dermis was linked to inflammation and fibrotic scar formation (Figure 6). Overall, this phage-cocktail dressing synergistically integrates antibacterial defense, immunomodulation, and neuropeptide signaling to drive regenerative healing (Figure 7).

**FIGURE 7:**
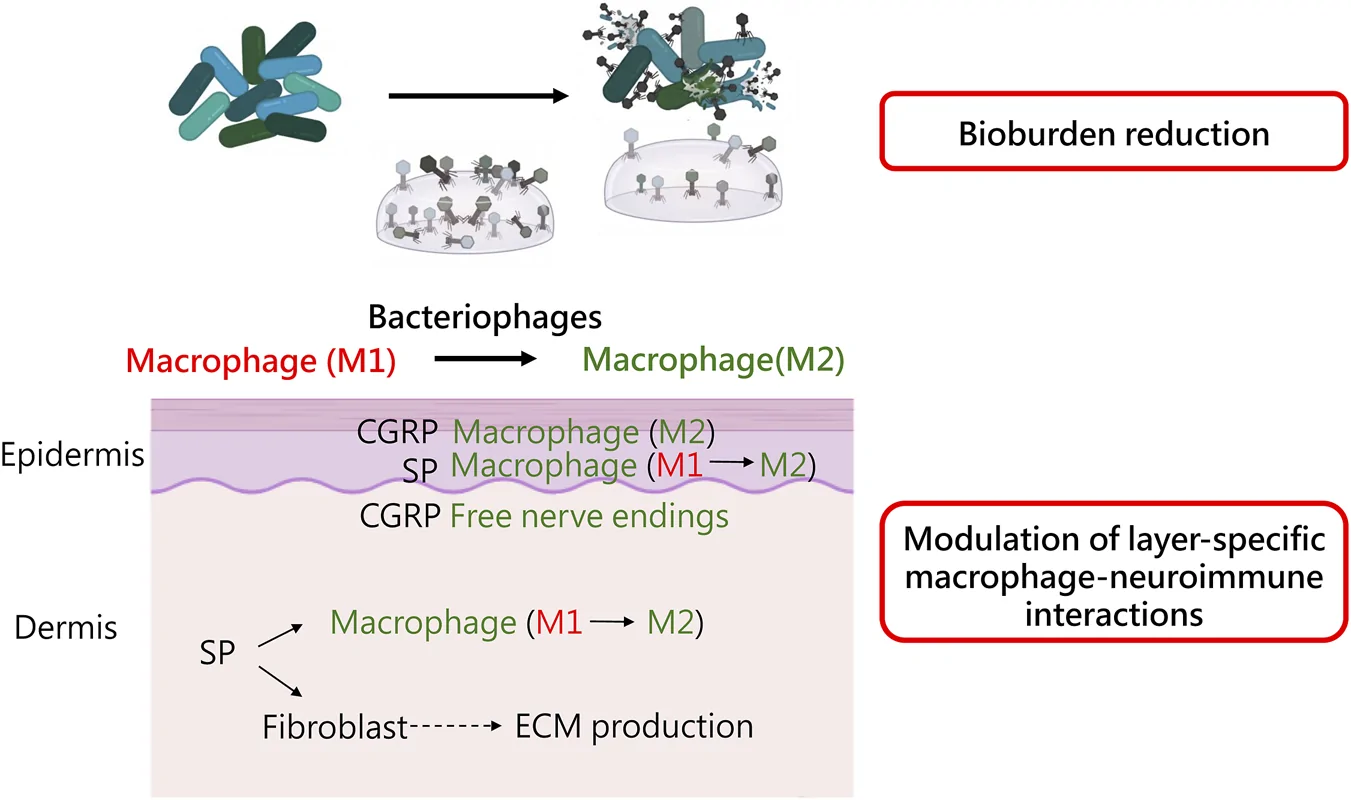
Schematic diagram illustrating the proposed mechanisms of the sprayable phage-cocktail dressing in promoting diabetic wound healing by reducing bioburden to prevent ulcer formation and modulating layer-specific neuroimmune-macrophage interactions. This dressing reduces bioburden to prevent ulcer formation, promotes M2 macrophage polarization, and modulates neuroimmune interactions cues, macrophage, SP and CGRP, to enhance re-epithelialization and ECM remodeling. Specifically, it upregulates epidermal SP to drive the M1-to-M2 macrophage transition and increases epidermal CGRP expression to maintain M2 macrophages in the epidermis, thereby promoting re-epithelialization. In the dermis, elevated SP levels support extracellular matrix (ECM) remodeling. Together, these effects contribute to tissue regeneration within the wound microenvironment.

The phage-cocktail dressing promoted M2 macrophage polarization. Macrophages play a critical role in inflammation and tissue repair during wound healing. They transition from a pro-inflammatory M1 phenotype, present in the early stages of repair, to an anti-inflammatory M2 phenotype, which promotes tissue repair and regeneration (Hesketh et al., 2017). In diabetic wounds, macrophage dysfunction, including delayed or insufficient M2 polarization, leads to persistent inflammation and impaired healing. Our study found that the phage-cocktail dressing promoted M2 macrophage polarization, which may contribute to better outcomes in wound healing by reducing inflammation and supporting tissue repair. Indeed, quantitative analysis confirmed the dressing significantly reduced M1 macrophages to 1.5% while increasing M2 macrophages to 12.9% (Figure 4E). Additionally, macrophages also regulate the proliferation, differentiation, and migration of keratinocytes, fibroblasts, and endothelial cells, facilitating neovascularization and wound closure (Aitcheson et al., 2021). Enhancing macrophage function could therefore help wound closure and minimize scar formation and accelerate the wound healing process.

The therapeutic efficacy of the phage-cocktail hydrogel is closely correlated with the modulation of neuropeptide signaling within the diabetic wound microenvironment. SP facilitates M1-to-M2 macrophage polarization and dermal ECM remodeling by downregulating the NF-κB/NLRP3 pathway (Zhou et al., 2023) and stimulating keratinocyte and fibroblast migration (Kim et al., 2018). While impaired SP signaling typically disrupts diabetic wound healing (Yang et al., 2022), our phage-cocktail dressing was found to upregulate SP levels and M2 macrophage abundance. Specifically, SP H-scores significantly increased to 171.3 in the epidermis and 163.7 in the dermis (Figure 5B). By suppressing pro-inflammatory cytokines (TNF-α, IL-1β) and MMP-9, this restoration promotes granulation tissue formation and matrix remodeling (Kant et al., 2015). Ultimately, the hydrogel addresses core diabetic dysfunctions in part through its regulatory effects on the SP and CGRP signaling axis, thereby driving immunomodulation, re-epithelialization, and regenerative healing (Figure 7).

Parallelly, CGRP exhibited a layer-specific functional divergence. In the epidermis, the phage-cocktail dressing significantly elevated CGRP expression, yielding a high epidermal CGRP H-score of 151.0 (Figure 5B), which correlated with increased M2 macrophage abundance and improved epithelial regeneration. Mechanistically, CGRP acts via the RAMP1 receptor to modulate immune responses and promote M2 polarization (Lu et al., 2024); these M2-derived cytokines subsequently support keratinocyte maturation and maintain tissue homeostasis (Savitri et al., 2022).

However, CGRP expression in the deeper dermis appears linked to pathological outcomes. In our study, dermal CGRP was primarily localized within scar tissue, consistent with its elevated expression in both keloids (Suarez et al., 2015) and the reticular dermis of hypertrophic burn scars, where it correlates with heightened neurogenic inflammation, pain, and pruritus (Kwak et al., 2014). Indeed, scar tissues exhibited a significantly higher CGRP H-score (162.0) but lower SP H-score (100.7) than non-scar regions (Figure 6D). Mechanistically, CGRP has been shown to drive pulmonary fibrosis by inducing myofibroblast differentiation and ECM production via Smad-dependent TGF-β and Wnt signaling pathways (Kayalar and Oztay, 2022). Given the central role of myofibroblasts in cutaneous repair (Younesi et al., 2024), we posit that CGRP may similarly orchestrate fibrotic responses in the dermis through peripheral nerve sensitization and mast cell activation. Furthermore, since nearly 50% of diabetic patients suffer from peripheral neuropathy (Feldman et al., 2019), the dysregulation of CGRP-mediated signaling observed here may represent a critical link between impaired neural feedback and the fibro-inflammatory nature of diabetic wounds. Future research is warranted to further elucidate these spatial neuropeptide dynamics and verify the direct causal pathways. While further mechanistic validation is needed, our data suggests that the hydrogel holds potential to transition from chronic inflammation to functional regenerative repair.

Phage therapy potentially contributing to chronic diabetic wound healing through preventing biofilm formation and the potential modulation of microbiomes. Biofilm-associated pathogens release endotoxins like lipopolysaccharide (LPS), driving persistent inflammation and delaying repair (Kim et al., 2020). Phages can suppress LPS-induced immune responses (Miernikiewicz et al., 2016) and enhance phagocytic activity (Chhibber et al., 2013). Phage therapy disrupts biofilms and reduces pathogens, helping wound progression toward complete, reinfection-free recovery (Kovacs et al., 2024). A balanced wound microbiome is essential for proper healing. Chronic wounds exhibit reduced bacterial diversity, negatively affecting immune responses and tissue integrity (Huang et al., 2022). Unlike broad-spectrum antibiotics, phage therapy selectively targets pathogenic bacteria while potentially preserving beneficial skin commensals, like *Staphylococcus epidermidis* enhance wound repair through immune modulation (Patel et al., 2022); a similar precision is seen in IBD therapy to selectively reduce adherent-invasive *E. coli* (AIEC) without disrupting gut homeostasis (Cinquerrui et al., 2018). Due to the limited surface area of the skin wounds, microbial diversity analysis could not be performed in this study; however, maintaining wound microbiome homeostasis likely remains a significant advantage of phage therapy that warrants future microbiological investigation.

In conclusion, while sharp debridement disrupts mature biofilms, bacterial recolonization and compromised immunity persist as critical hurdles (Sussman, 2023); although conventional antimicrobial dressings reduce bioburdens, they fail to restore impaired host defenses (Sussman, 2023). Bridging this gap, our sprayable phage-cocktail hydrogel not only reduces bacterial bioburden but also restores regenerative healing in obese-diabetic mice by modulating the SP/CGRP neuroimmune signaling axis, reflecting its potential role in modulating neuroimmune dynamics. Specifically, upregulated SP drives M1-to-M2 macrophage polarization to facilitate dermal ECM remodeling, while epidermal CGRP sustains M2 macrophages to enhance re-epithelialization. By modulating these layer-specific neuropeptide dynamics and preventing dermal CGRP-mediated fibrotic scarring, this hydrogel effectively transitions chronic inflammation toward functional tissue repair. These results offer a promising therapeutic strategy for the clinical management of complex diabetic ulcers.

## Data Availability

The original contributions presented in the study are included in the article/supplementary material, further inquiries can be directed to the corresponding authors.
